# Lower Abdominal and Periumbilical Pain in a Patient With Focal Nodular Hyperplasia of the Liver: A Case Report and Literature Review

**DOI:** 10.7759/cureus.87182

**Published:** 2025-07-02

**Authors:** Ron Bergmann, Stefan Wildi, Joerg Wydler, Christian Booz, Thomas J Vogl

**Affiliations:** 1 Department of General Practice, Medvadis Ärtzezentrum Schlieren, Zurich, CHE; 2 Department of Visceral, Thoracic and Vascular Surgery, Stadtspital Waid, Zurich, CHE; 3 Department of Visceral, Thoracic and Vascular Surgery, Stadtspital Triemli, Zurich, CHE; 4 Department of Radiology and Nuclear Medicine, University Clinic Frankfurt am Main, Frankfurt am Main, DEU

**Keywords:** fnh, gastrointestinal surgery, general medicine, general practice, hepatic surgery, hepatobiliary surgery

## Abstract

Focal nodular hyperplasia (FNH) is the second most common benign liver tumor, primarily affecting women of childbearing age. It is typically asymptomatic and carries no risk of malignant transformation. While conservative management is usually preferred, diagnostic uncertainty or symptomatic cases may require intervention. Here, we present the case of a 23-year-old female with chronic periumbilical and lower abdominal pain. Extensive laboratory and imaging studies, including contrast-enhanced CT and MRI, revealed no abnormalities. Diagnostic laparoscopy was performed, which identified a superficially located, multilobulated mass on liver segment IVb. The patient opted for laparoscopic atypical liver segment resection, and histopathological analysis confirmed FNH. Postoperatively, the patient experienced symptom relief without complications. While most FNH cases are incidental and do not require intervention, this case highlights the diagnostic challenges and management of symptomatic lesions. Imaging, particularly contrast-enhanced MRI, is crucial to diagnosis, although biopsy may be necessary in atypical cases. Surgical resection is generally reserved for cases with diagnostic uncertainty, progressive lesion growth, or persistent symptoms. Minimally invasive options, such as transarterial chemoembolization or radiofrequency ablation, may serve as alternative treatment approaches. This case underscores the importance of individualized decision-making in the management of FNH. Although conservative management is typically the standard approach, surgical resection may be justified in symptomatic patients or when the diagnosis is unclear. Minimally invasive therapies offer promising alternatives in select cases.

## Introduction

Focal nodular hyperplasia (FNH) was first described in 1958 by the pathologist Hugh Edmondson and is the second most common benign liver tumor after hepatic hemangioma. It occurs most frequently in women of childbearing age, with a female-to-male ratio of about 8:1, affecting up to 3% of the general population [1] with a prevalence of approximately 0.8% in adult autopsy studies [2]. Typically, it appears as a single lesion, although multiple lesions or concurrent hepatic adenomas may also occur. No case of malignant transformation has been documented to date. Complications are rare but may include rupture and hemorrhage, especially in lesions exceeding 5 cm in diameter. However, these complications occur more frequently in hepatic adenomas [3].

FNH is thought to arise from vascular malformations that alter intrahepatic blood flow, leading to a localized hyperplastic response of otherwise normal hepatocytes [4]. Cytologically, the contained hepatocytes are free of atypia and thus indistinguishable from "normal" hepatocytes, further suggesting the benign nature of this tumor [5]. An association between the use of oral contraceptives and FNH has been proposed; however, evidence-based data remains insufficient. Some studies indicate that daily use of oral contraceptives may be associated with larger lesion size [6]. Furthermore, female patients, regardless of oral contraceptive use, tend to develop larger lesions than male patients [7].

## Case presentation

**Figure 1:**
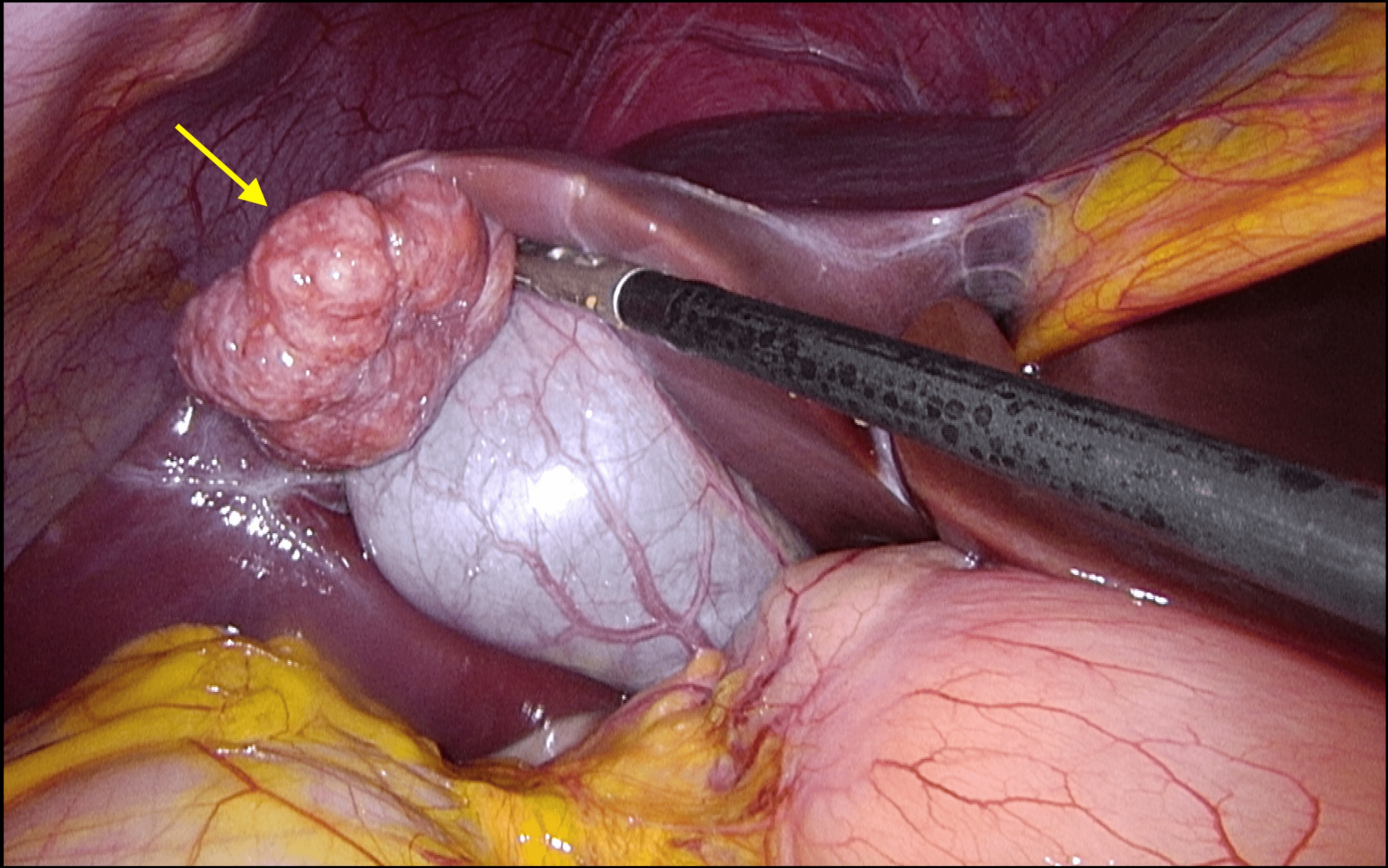
Intraoperative finding of a superficially located, multilobulated mass on liver segment IVb (yellow arrow)

**Figure 2:**
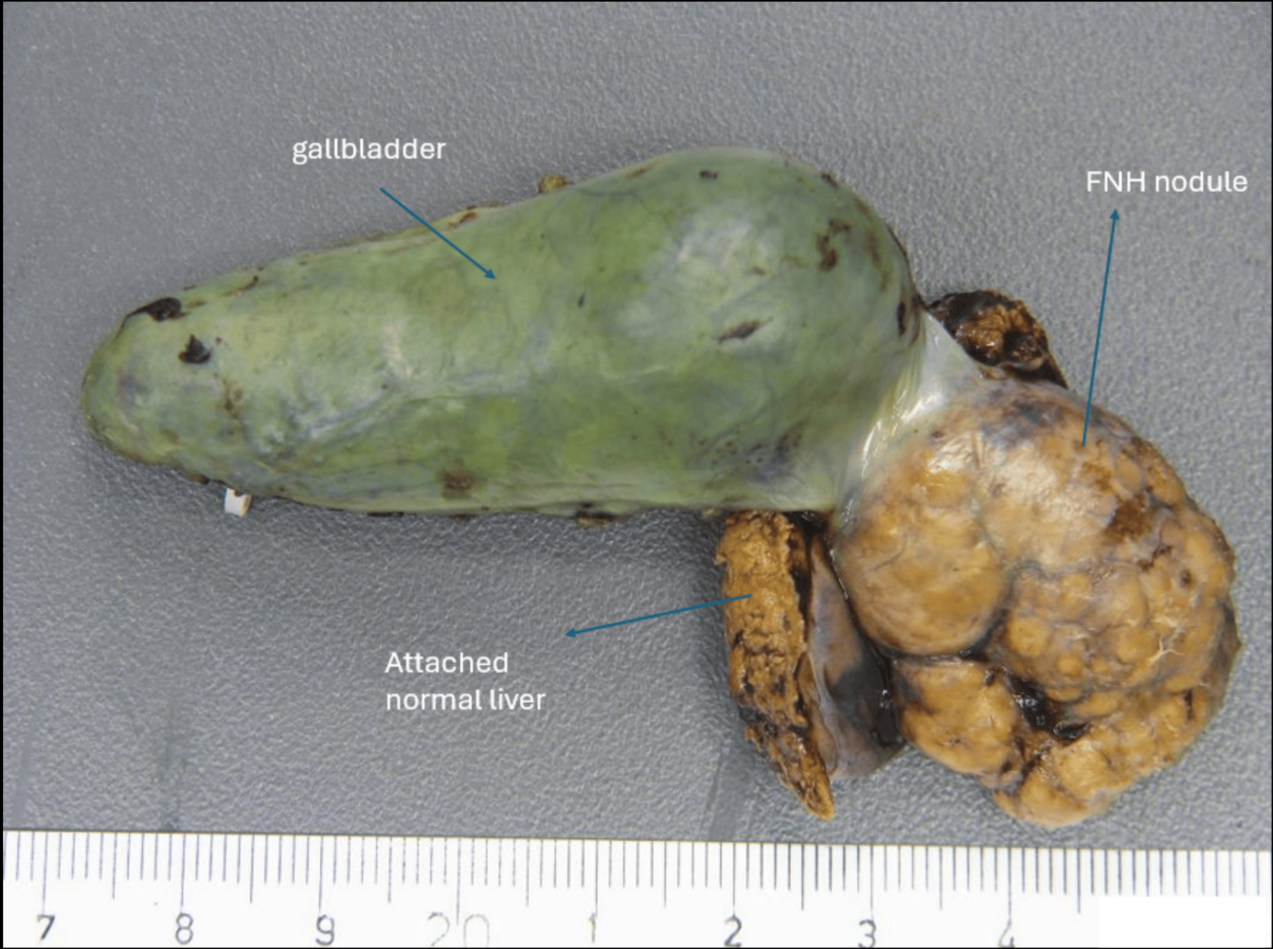
Resected liver specimen FNH: focal nodular hyperplasia

A 23-year-old woman presented to her general practitioner with several months of chronic periumbilical and lower abdominal pain. Physical examination revealed mild tenderness in the right upper quadrant. Laboratory tests were unremarkable. Imaging studies, including contrast-enhanced MRI and CT scans of the abdomen and pelvis, as well as upper and lower endoscopy and a comprehensive gynecological evaluation, did not reveal any abnormalities. Given her history of an appendectomy, a diagnostic laparoscopy was performed to rule out adhesions. No adhesions were found. However, a superficially located, multilobulated mass was discovered on liver segment IVb (Figure 1). Examination of the small intestine and pelvic organs revealed no further pathologies. Intraoperatively, FNH or hepatocellular adenoma were considered as differential diagnoses. No additional procedures were performed at that time. The incidental nature of the lesion and the possibility that it might not be responsible for the patient’s symptoms were discussed postoperatively. The patient was informed that resection might not alleviate her pain but would reduce long-term diagnostic uncertainty and the need for further follow-up. In February 2022, laparoscopic atypical resection of segment IVb was performed at the Triemli site of the City Hospital Zurich. The procedure was uneventful, with no postoperative complications. Pain was managed with patient-controlled analgesia, and the patient was discharged two days later. The resected specimen contained a well-circumscribed, multilobulated lesion measuring 35 mm in diameter (Figure 2). Histopathological analysis confirmed FNH.

**Figure 3:**
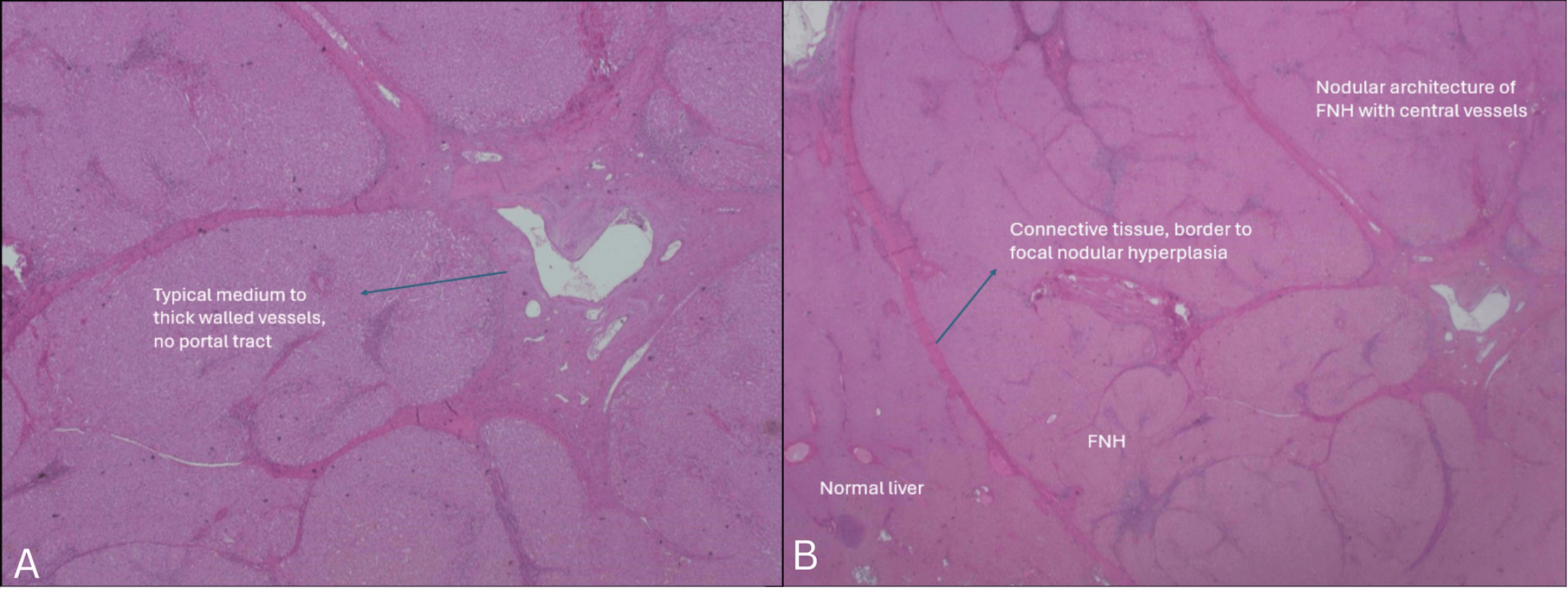
Histological specimen, H&E stain. (A) Malformed arteries embedded within the central scar. (B) Histological specimen showing the characteristic central scar of FNH, composed of fibrous connective tissue H&E: hematoxylin and eosin, FNH: focal nodular hyperplasia

**Figure 4:**
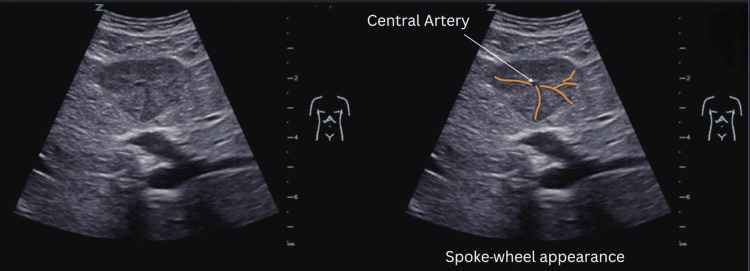
Native ultrasound showing spoke-wheel pattern with centrifugal radiating vessels originating from the central artery typical of FNH FNH: focal nodular hyperplasia

**Figure 5:**
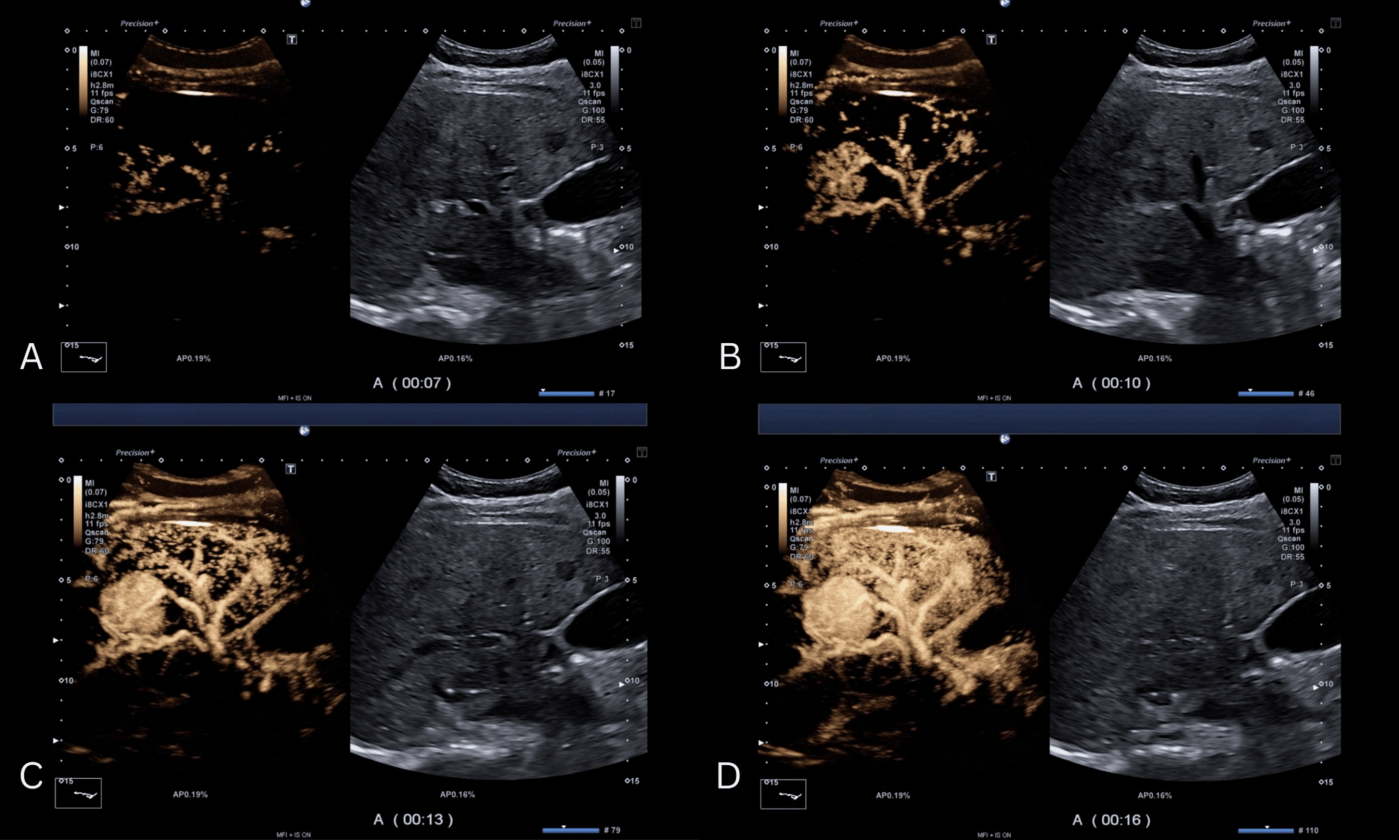
Sequential CEUS imaging demonstrating centrifugal contrast filling of branching arteries. (A–D) Time-lapse series showing progressive enhancement CEUS: contrast-enhanced ultrasound

**Figure 6:**
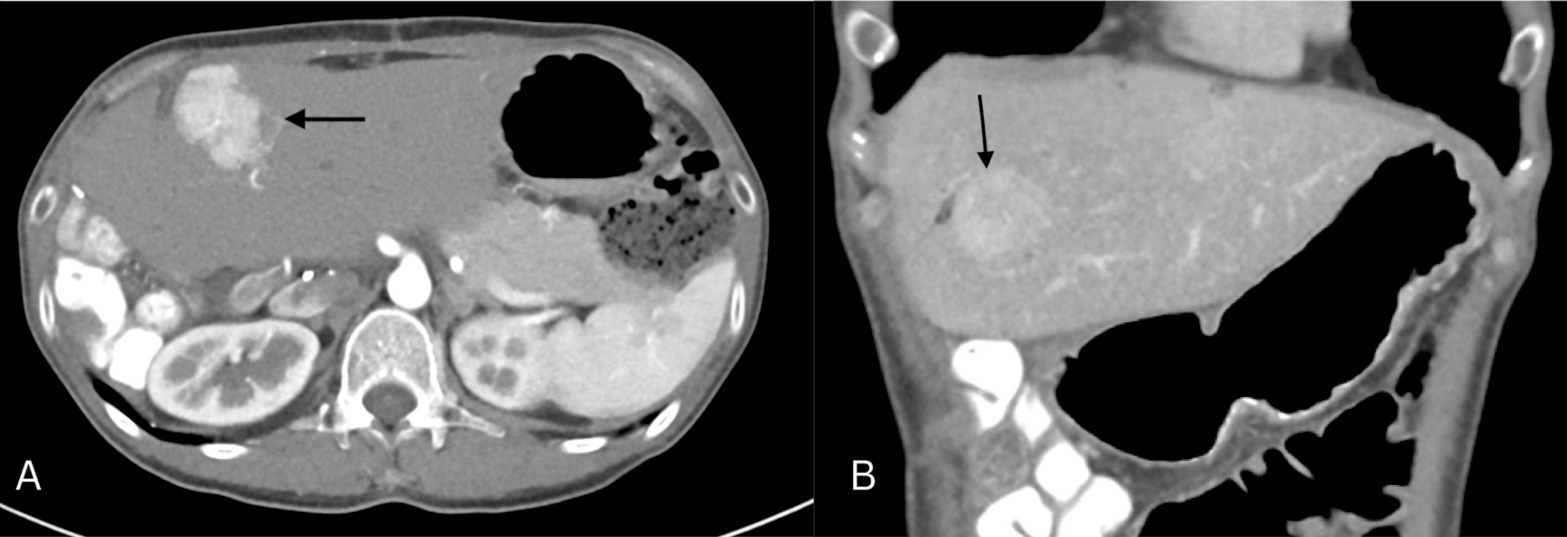
Contrast-enhanced CT of the liver, black arrows pointing at FNH. (A) Arterial phase: hyperdense lesion compared to surrounding parenchyma. (B) Venous phase: isoattenuation CT: computed tomography, FNH: focal nodular hyperplasia

**Figure 7:**
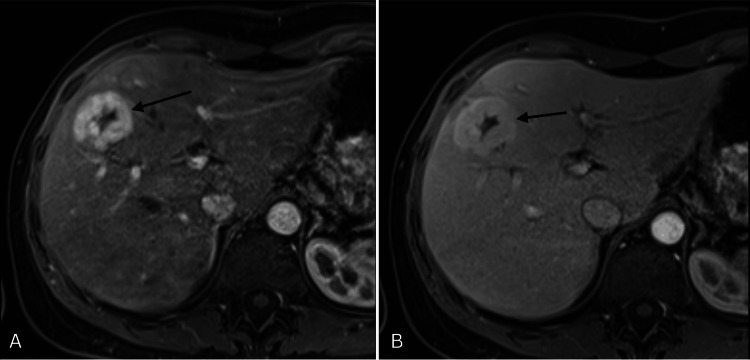
Contrast-enhanced MRI of the liver, black arrows pointing at FNH. (A) Arterial phase: intense enhancement of the lesion. (B) Venous phase: attenuation of contrast uptake MRI: magnetic resonance imaging, FNH: focal nodular hyperplasia

One of the hallmark features of FNH, the central stellate scar, was evident, composed of fibrous connective tissue with aberrant arteries (Figures 3A-3B). Branches typically radiate centrifugally from the central artery, resembling the shape and appearance of a “spoke-wheel” (Figure 4). Contrast-enhanced ultrasound (CEUS) imaging demonstrates a typical centrifugal filling pattern of the branching arteries. As seen in the time-lapse sequence (Figure 5A-5D), early enhancement begins centrally and progressively extends peripherally, consistent with the vascular architecture of FNH. Contrast-enhanced axial imaging using CT and MRI can often confirm the diagnosis of FNH without the need for further intervention. During the arterial phase of CT, FNH typically demonstrates strong enhancement due to its rich vascularization, appearing hyperdense compared to the surrounding liver tissue (Figure 6A). Isoattenuation is observed during the venous phase (Figure 6B). On abdominal MRI, strong enhancement is evident throughout the arterial phase (Figure 7A), followed by attenuation during the venous phase (Figure 7B). In the present case, however, contrast-enhanced CT and MRI performed prior to resection did not reveal any abnormalities. In follow-up telephone calls over the subsequent months, the patient reported significant relief of symptoms.

## Discussion

First and foremost, it is worth noting that the majority of FNH cases are asymptomatic and do not require any intervention. However, controversy remains regarding the indications for intervention in symptomatic cases or when diagnostic uncertainty persists [8]. Two primary questions guide the clinical approach to FNH: First, is the lesion symptomatic? Second, can a confident diagnosis be made noninvasively?

Accurate differentiation between benign and malignant focal liver lesions is crucial, especially in diagnostically ambiguous cases. CEUS has proven valuable for characterizing small lesions, offering dynamic vascular imaging. Table 1 illustrates the typical CEUS enhancement patterns in benign and malignant liver lesions, supporting radiologic assessment when standard imaging is inconclusive [9].

**Table 1:** CEUS-based enhancement patterns of benign and malignant liver lesions CEUS: contrast-enhanced ultrasound [9]

Lesion	Phase	Typical features	Additional features
Hemangioma	Arterial phase	Peripheral nodular enhancement	Small lesion: complete, rapid centripetal enhancement
Hemangioma	Portal venous phase	Partial/complete centripetal fill in	-
Hemangioma	Late phase	Incomplete or complete enhancement	Nonenhancing regions
Hemangioma	Post-vascular phase	Iso/slightly hypo-enhancing	Nonenhancing regions
FNH	Arterial phase	Hyperenhancing from the center, complete, early	Spoke-wheel arteries
FNH	Portal venous phase	Hyperenhancing	Unenhanced central scar
FNH	Late phase	Iso/hyperenhancing	Unenhanced central scar
FNH	Post-vascular phase	Iso/slightly hyper- or hypoenhaning	-
Hepatocellular adenoma	Arterial phase	Hyperenhancing, complete	Nonenhancing regions
Hepatocellular adenoma	Portal venous phase	Isoehnancing	Hyperenhancing
Hepatocellular adenoma	Late phase	Isoehnancing	Slightly hypoenhaning
Hepatocellular adenoma	Post-vascular phase	-	Nonenhancing regions
Focal fatty infiltration	Arterial phase	Isoehnancing	-
Focal fatty infiltration	Portal venous phase	Isoehnancing	-
Focal fatty infiltration	Late phase	Isoehnancing	-
Focal fatty infiltration	Post-vascular phase	Isoehnancing	-
Focal fatty sparing	Arterial phase	Isoehnancing	-
Focal fatty sparing	Portal venous phase	Isoehnancing	-
Focal fatty sparing	Late phase	Isoehnancing	-
Focal fatty sparing	Post-vascular phase	Isoehnancing	-
Abscess	Arterial phase	Peripheral enhancement, no central enhancement	Hypoenhancing rim; enhanced septa; hyperenhanced liver segment
Abscess	Portal venous phase	Hyper-/isoehnancing rim, no central enhancement	Enhanced septa; hyperenhanced liver segment
Abscess	Late phase	Hypoenhancing rim, no central enhancement	Hypoenhancing rim
Abscess	Post-vascular phase	Hypoenhancing rim	-
Simple cyst	Arterial phase	Nonenhancing	-
Simple cyst	Portal venous phase	Nonenhancing	-
Simple cyst	Late phase	Nonenhancing	-
Simple cyst	Post-vascular phase	Nonenhancing	-
Metastasis	Arterial phase	Rim-enhancement	Complete enhancement; hyperenhancement; nonenhancing regions
Metastasis	Portal venous phase	Hypoenhancing	Nonenhancing regions
Metastasis	Late phase	Hypo/nonenhancing	Nonenhancing regions
Metastasis	Post-vascular phase	Hypo/nonenhancing	Nonenhancing regions
HCC	Arterial phase	Hyperenhancing	Nonenhancing regions
HCC	Portal venous phase	Isoehnancing	Nonenhancing regions
HCC	Late phase	Hypo/nonenhancing	Nonenhancing regions
HCC	Post-vascular phase	Hypo/nonenhancing	Nonenhancing regions
Cholangiocarcinoma	Arterial phase	Rim-like hyperenhancement, central hypoenhancement	Nonenhancing regions; inhomogeneous hyperenhancement
Cholangiocarcinoma	Portal venous phase	Hypoenhancing	Nonenhancing regions
Cholangiocarcinoma	Late phase	Hypo/nonenhancing	Nonenhancing regions
Cholangiocarcinoma	Post-vascular phase	Hypo/nonenhancing	Nonenhancing regions

Due to the lack of malignant potential, conservative management is generally recommended when diagnostic certainty exists and no comorbid hepatic pathology is present. Surgical or minimally invasive procedures should be considered only in cases of diagnostic ambiguity, progressive lesion growth, or persistent symptoms despite conservative care. Symptomatology is thought to arise from capsular stretching and may manifest as right upper quadrant or epigastric pain, nausea, or vague discomfort. A palpable mass is rare, and abnormal laboratory findings such as elevated liver enzymes or tumor markers are uncommon [10]. Diagnostic algorithms typically include ultrasound, CT, and MRI, with relatively low diagnostic sensitivity attributed to conventional ultrasound [11]. Doppler ultrasound may demonstrate centrally arising arteries extending peripherally. CEUS is superior to axial imaging in detecting small FNH lesions < 3 cm in size, with a reported sensitivity of 93% and a specificity of 100% [12]. This makes CEUS the diagnostic modality of choice for small lesions.

A 2007 study demonstrated that the combination of contrast-enhanced CT and MRI enhanced diagnostic sensitivity, with reported rates of 60% for CT and 77% for MRI [11]. A 2015 systematic review, which included 309 patients, reported a sensitivity of 91-100% and a specificity of 87-100% for MRI with a hepatobiliary contrast agent [13].

In MRI, early arterial enhancement with a centrifugal filling pattern can be observed in both FNH and hepatic adenomas [14]. The treatment of these two lesions differs significantly, as they vary considerably in their potential for hemorrhage or malignant transformation. The use of hepatobiliary contrast agents (hepatocyte-specific contrast agents, HSCA) provides an additional tool to increase diagnostic sensitivity. These are typically gadolinium-based agents such as gadoxetic acid (Primovist®) or gadopentetate dimeglumine. HSCA allows for the differentiation between hepatic and extrahepatic lesions, such as metastases or hemangiomas [15]. According to EASL guidelines, FNH can be diagnosed with 98% specificity and 70% sensitivity if no liver disease or lab abnormalities are present [12]. Diagnostic imaging should show homogeneous lesions, a central scar, arterial phase enhancement without washout, septae, and no capsule.

In cases of atypical appearance, diagnostic ambiguity, or when a malignant process cannot be safely excluded, a biopsy may be necessary. This may be the case with atypical FNH, which accounts for about 20% of all FNH and FNH-like lesions, as the absence of a central scar or other hallmark features may obscure the diagnosis and raise concern for malignancy. In such cases, percutaneous or laparoscopic biopsy is warranted. Alternatively, resection allows for both diagnosis and treatment.

A retrospective single-center study from 2012 [16] involving 185 patients diagnosed with FNH compared the outcomes of 78 patients who underwent elective hepatectomy with 107 patients who were only observed, with a median follow-up of 113 months. 92% of patients in the surgical arm reported symptom relief, compared to 12% in the non-surgical arm, who reported persistent symptoms.

A 2014 review article, which analyzed a total of 14 studies and included 885 patients, concluded that diagnostic uncertainty remains the primary indication for surgical intervention, as there are reports of patients experiencing spontaneous reduction or even complete remission of symptoms when treated conservatively [17]. Conversely, in one of the studies reviewed in the article, surgical resection was performed due to an increase in the lesion size [15]. A similar conclusion was reached in a retrospective observational study involving 48 patients from 2019 [18]. The authors used ultrasound (51%), CT (100%), and MRI (90%) and identified diagnostic uncertainty as the most important indicator for surgical resection. Some patients had radiological findings consistent with FNH, but biopsy results indicated a malignant comorbidity that justified a surgical approach. The conclusion was that FNH can be diagnosed through imaging; however, suspicious findings indicating HCC or another malignancy still justify surgical intervention.

When choosing a surgical procedure, a careful assessment of the benefits and risks is required, especially since there are few controlled studies on this topic. Some authors even warn of unacceptably high morbidity and mortality rates associated with open or laparoscopic surgery for FNH [1]. Intra- and postoperative complications of surgical intervention can range from venous thromboembolism, ileus, intra-abdominal abscess, pleural effusion, bleeding, and hepatobiliary fistulas to acute liver failure and, not least, cosmetically significant scarring. Large lesions greater than 5 cm are typically considered for surgical treatment; conversely, lesions can also be treated minimally invasively first to reduce their size before surgical intervention. Minimally invasive procedures are used, for example, for smaller lesions less than 5 cm in diameter, in inoperable patients, or those with contraindications for surgical intervention. Among the minimally invasive options are procedures such as transarterial chemoembolization (TAE), radiofrequency ablation (RFA), and thermal or microwave ablation.

TAE can be performed using various substances, such as polyvinyl alcohol [19], trisacryl gelatin microspheres [20], bleomycin, or bleomycin in combination with iodized oil (Lipiodol®) [21,22]. One of this article's authors published a study in 2006 involving four female patients with FNH who underwent superselective TAE with polyvinyl alcohol [19]. The indication for intervention was either size progression or a symptomatic lesion, with a one-year follow-up showing a reduction in size in 50% of patients and complete remission in the remaining 50%.

RFA or microwave ablation are thermal ablation procedures and also represent valid options. To date, several studies have shown promising results with few intra- or post-interventional complications [23-25]. Zhang et al. conducted a comparative study involving 82 patients with FNH measuring less than 5 cm in maximum diameter, of whom 39 underwent thermal ablation and 43 underwent surgical treatment [25]. The ablated group had a statistically significantly shorter procedure duration, less blood loss, and less destruction of healthy liver parenchyma. Furthermore, lower costs and a shorter recovery time were observed compared to surgical intervention.

## Conclusions

We report a case of FNH in a 23-year-old female presenting with lower abdominal pain. Despite extensive imaging and endoscopic evaluation, no abnormalities were detected preoperatively. A diagnostic laparoscopy revealed a hepatic lesion consistent with FNH, which was subsequently resected. Histopathology confirmed the diagnosis, and the patient experienced resolution of symptoms.

This case underscores the importance of individualized management in FNH. While asymptomatic lesions should be managed conservatively, intervention may be warranted in symptomatic cases or when imaging cannot rule out malignancy. Surgical resection remains a valid therapeutic modality, especially for large lesions, but carries higher risks, costs, and longer hospital stays. Minimally invasive procedures such as TAE or RFA represent promising, lower-risk, cost-effective alternatives that result in less peri-interventional trauma and shorter recovery times.
